# Development and Stability Studies of Novel Liposomal Vancomycin Formulations

**DOI:** 10.5402/2012/636743

**Published:** 2012-01-26

**Authors:** Krishna Muppidi, Andrew S. Pumerantz, Jeffrey Wang, Guru Betageri

**Affiliations:** ^1^Department of Pharmaceutical Sciences, College of Pharmacy, Western University of Health Sciences, 309 E. 2nd Street, Pomona, CA 91766, USA; ^2^Division of Infectious Disease, Department of Internal Medicine, College of Osteopathic Medicine of the Pacific, Western University of Health Sciences, Pomona, CA 91766, USA

## Abstract

A promising strategy to improve the therapeutic efficiency of antimicrobial agents is targeted therapy. Although vancomycin has been considered a gold standard for the therapy of MRSA pneumonia, clinical failure rates have also been reported owing to its slow, time-dependent bactericidal activity, variable lung tissue penetration and poor intracellular penetration into macrophages. Liposomal encapsulation has been established as an alternative for antimicrobial delivery to infected tissue macrophages and offers enhanced pharmacodynamics, pharmacokinetics and decreased toxicity compared to standard preparations. The aim of the present work is to prepare vancomycin in two different liposomal formulations, conventional and PEGylated liposomes using different methods. The prepared formulations were optimized for their particle size, encapsulation efficiency and physical stability. The dehydration-rehydration was found to be the best preparation method. Both the conventional and PEGylated liposomal formulations were successfully formulated with a narrow particle size and size distribution and % encapsulation efficiency of 9 ± 2 and 12 ± 3, respectively. Both the formulations were stable at 4°C for 3 months. These formulations were successfully used to evaluate for their intracellular killing of MRSA and *in vivo* pharmacokinetic and bio-distribution studies.

## 1. Introduction

**Figure 1:**
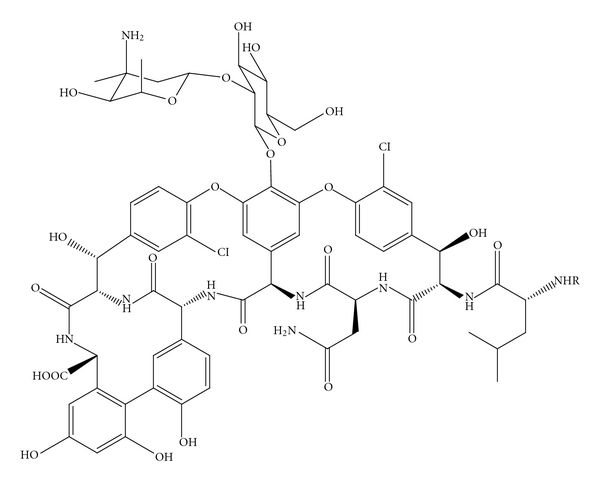
Chemical structures of vancomycin (R = CH_3_) and norvancomycin (R = H).

Methicillin-resistant *Staphylococcus aureus* (MRSA) has become an increasingly important etiology of pneumonia both in healthcare and community settings. Although previously considered as a nosocomial pathogen, in recent years it has been diagnosed with increased incidence at hospital admission [1]. *S. aureus* causes a wide spectrum of mild to severe infections both in humans and animals [2]. Several factors contribute to the persistence and recurrence of these infections, but an important feature is the ability of the bacteria to invade and survive inside the phagocytic cells [3]. Vancomycin (Figure 1) has been considered a gold standard for the therapy of MRSA infections yet is poorly concentrated within human macrophages [4, 5]. Vancomycin is a branched, tricyclic, glycosylated, and nonribosomal peptide produced by *Streptomyces orientalis *[6]. It produces antibacterial activity without requiring the penetration of the lipid membrane [7]. Vancomycin binds with high-affinity *D*-alanine-*D*-alanine C-terminus of late peptidoglycan precursors to prevent transpeptidation required for synthesis of bacterial cell walls [8]. Clinical failures with vancomycin against methicillin-resistant *Staphylococcus aureus* (MRSA) infections have challenged its standing as a first-line antimicrobial [9]. The use of antibiotic delivery systems with capacity for selective distribution in phagocytic cells is an important resource in improving antibiotic therapy against intracellular infections. Liposomes have been widely considered as potential drug delivery systems ever since the published observation of Bangham and coworkers [10]. They are currently used as unique drug carriers in several cosmetic and pharmaceutical industries. Liposomes are colloidal vesicles ranging from few nanometers to several micrometers in diameter with one or several lipid bilayers surrounding an inner aqueous compartment [11, 12]. Liposomes are biodegradable, biocompatible, nontoxic, and nonimmunogenic [13]. They can entrap a wide variety of therapeutic drugs including antimicrobial, and anticancer drugs [14]. Hydrophilic drugs can be encapsulated in the aqueous core, and lipophilic drugs can be entrapped in the lipid bilayers [13]. Several factors such as aqueous volume, membrane rigidity, surface area, and preparation methods of liposomes have influence on the encapsulation efficiency of liposomes [15, 16].

When conventional liposomes are administered *in vivo*, they are rapidly cleared from the blood circulation by monocytes and macrophages and are thus accumulated in the organs of reticuloendothelial system (RES), especially the liver and spleen [17, 18], which makes them to be a more relevant target for intracellular infections localized in these tissues [19]. Unlike conventional liposomes, PEGylated liposomes are able to avoid rapid hepatosplenic uptake. Owing to the biocompatible PEG coating on their surface, PEGylated liposomes delay opsonization and hence allow for relatively longer blood circulation times, thus creating possibilities to target intercellular pathogens and infected macrophages outside the liver and spleen [20]. PEGylated liposomes after long-term circulation in blood extravasate in the infected tissues and eventually end up in deep-seated tissue macrophages, thus acting as site-specific drug delivery systems [14].

Vancomycin, being highly hydrophilic in nature, is not an ideal candidate drug for liposome encapsulation. In the present work, several methods have been attempted for this challenging endeavor. In general, entrapment of hydrophilic drugs using the passive loading film hydration method [10] is not efficient. The pH gradient method leading to quantitative drug loading of vesicles through inward active drug diffusion is limited to drugs of small molecular weight and certain structural characteristics such as charge property [21]. On the other hand, the dehydration-rehydration vesicle (DRV) method [15] can entrap drugs regardless of their molecular size, but the size of liposomes is relatively large. However, smaller-sized liposomes entrapping vancomycin with high efficiency can be prepared using a modified dehydration-rehydration method [22, 23]. In the modified DRV method, preformed empty small unilamellar liposomes are subjected to dehydration in the presence of appropriate ratio of sugar to lipid, and then the drug destined to entrapment is added to empty liposomes in solution form, resulting in increased entrapment compared to other conventional methods.

In this study, our goal was to develop novel vancomycin liposomal formulations and characterize their stability profiles.

## 2. Materials and Methods

### 2.1. Materials

For the preparation of liposomal formulation, 1, 2-distearoyl-*sn*-glycero-3-phosphocholine (DSPC) and MPEG-2000-DSPE [N-(Carbonyl-methoxypolyethyleneglycol 2000)-1,2 distearoyl-*sn-*glycero-3-phosphoethanolamine] were obtained from Genzyme Pharmaceuticals (Cambridge, MA). Cholesterol, vancomycin hydrochloride, sodium chloride, sucrose, and trifluoroacetic acid were obtained from Sigma Chemicals (St. Louis, MO). Norvancomycin (Figure 1) was obtained from Northern China Pharmaceutical Corporation (Shijiazhuang, Hebei, China). Potassium chloride and dibasic sodium phosphate were obtained from Baker (Philipsburg, NJ). Monobasic potassium phosphate was procured from Fisher Scientific (Houston, TX). HPLC-grade chloroform, acetonitrile, and methanol were obtained from EMD Chemicals (Gibbstown, NJ). Nanopure water was used for all experiments.

### 2.2. Preparation of Vancomycin Liposomes

The liposomes were initially prepared using the thin-film hydration method [10] and the ammonium sulfate gradient method [23], reported elsewhere. Using thin-film hydration method, briefly, required amounts of lipids and cholesterol were dissolved in chloroform, and a thin film was formed on the inner side of the round bottom flask, by evaporating the solvent under vacuum using a rotavapor. The lipid film formed was stored overnight in vacuum desiccator to eliminate traces of chloroform. The film was then hydrated at 58°C, above the *Tc* of DSPC, using 10 mL of PBS (pH-7.0) containing 50 mg of vancomycin. The formed liposomes were then sonicated using a probe sonicator in 5 cycles of 2 min each and were then serially sized by extrusion through 0.8, 0.4, and 0.2 *μ*m pore-sized polycarbonate membranes. The un-encapsulated drug was then separated using sepharose-4B column and the encapsulation efficiency was calculated spectrophotometrically at 280 nm. The liposomal fractions collected from the sepharose column were pooled and lyophilized after addition of suitable amount of sucrose.

Due to poor encapsulation efficiency of vancomycin using the thin-film hydration method, the liposomes were then prepared using a modified pH gradient method. Briefly the lipids were dissolved in a round-bottomed flask using chloroform, which was then removed by means of a rotary evaporator and by an overnight storage in a desiccator, thus allowing the formation of a thin lipid film. The lipid film was then hydrated with 1 mL of 250 mM ammonium sulfate solution and then subjected to 5 cycles of freezing (at −80°C) and thawing (with a water bath at 40°C), thus achieving a pH gradient with a homogenous acid environment in the intraliposomal aqueous compartments. Multilamellar vesicles were then subjected to extrusion through 0.8, 0.4, and 0.2 *μ*m pore-sized polycarbonate membranes. Unentrapped ammonium sulfate solution was removed by centrifugation at 4000 rpm for 15 min. The small unilamellar vesicles obtained after centrifugation were suspended in 5 mL of drug solution (50 mg/5 mL), and the resulting solution was allowed to equilibrate at room temperature for 3 hours. The unentrapped vancomycin was removed using a sepharose-4B column. The liposomes were passed through 100 mL of sepharose 4B column, and different fractions were collected and the liposomal fractions were pooled. The particle size and encapsulation efficiency of the prepared liposomal formulations were determined. The pooled liposomal fractions were subjected to lyophilization by adding suitable amount of sucrose as the cryoprotectant.

Due to poor encapsulation efficiency and poor stability of the prepared formulations using the above methods, it was decided to prepare subsequent formulations using a modified dehydration-rehydration method [24]. Both the conventional and PEGylated vancomycin liposomal formulations were prepared using the lipids DSPC, cholesterol, and MPEG-2000-DSPE in 3 : 1 : 0 and 3 : 1 : 0.02 molar ratios, respectively. The lipids were dissolved in 7 mL of chloroform and rotary-evaporated in a round-bottomed flask under vacuum, thus allowing the formation of a thin layer of lipid film. The lipid film formed was stored overnight in vacuum desiccator to eliminate trace of chloroform. The lipid film was then hydrated at 58°C, above the *Tc* of DSPC with 5 mL of nanopure water for 30 min to yield MLV liposomes. The MLVs thus formed were then sonicated for 2 min using a probe sonicator. The lipid dispersion was then frozen at −80°C and lyophilized overnight with the addition of sucrose to stabilize the liposomes during freeze-drying. DRVs were prepared by adding 1 mL of concentrated solution of vancomycin (50 mg/mL in PBS, pH 7.0) to the lyophilized liposomes and vortexing. The gel produced was allowed to stay at room temperature for 30 min and then diluted with 4 mL of PBS. The DRVs were then serially sized by extrusion through 0.8, 0.4, and 0.2 *μ*m pore-sized polycarbonate membranes. During the extrusion, the temperature of the extruder was maintained above 60°C using hot water circulation. Vancomycin-containing liposomes were separated from nonentrapped vancomycin by ultracentrifugation at 30,000 rpm (105,000 ×g) for 30 min, and the liposomal pellet obtained was washed once with 1 mL of PBS. The final pellet was resuspended in PBS, and the preparations were stored at 4°C.

### 2.3. Characterization of Liposomes

#### 2.3.1. Particle Size Measurement

The particle size of the liposomes was measured using a Nicomp submicron particle sizer (Model 370, Santa Barbara, California). Ten *μ*L of liposomal suspension was taken and suitably diluted to measure the mean diameter of DRVs. Gaussian distribution was used as the standard to measure the particle size.

#### 2.3.2. Determination of Encapsulation Efficiency (% EE)

Encapsulation efficiency was determined as the percentage of vancomycin incorporated into DRVs relative to the initial amount of drug added. The lipid vesicles were lysed using 10% Triton X-100 to determine the amount of drug present. Briefly, 25 *μ*L of liposomal suspension was added to 975 *μ*L of 10% Triton X-100 and vortexed for 5 min to facilitate lysis of the DRVs. The supernatant (100 *μ*L) was taken and used for HPLC analysis by addition of 5 *μ*L of norvancomycin (100 *μ*g/mL) as the internal standard. Encapsulation efficiency was calculated using:

(1) $$\text{\%}\;\text{Encapsulation}\;\text{efficiency}=\frac{A_{\text{DRVs}}}{A_{\text{sol}}}\times 100,$$

where *A*_DRVs_ is the amount of drug present in liposomes after lysis with 10% Triton X-100 and *A*_sol_ is the amount of drug added initially.

### 2.4. HPLC Method Development for the Quantification of Vancomycin Released from Liposomes

A sensitive, rapid, and accurate high-performance liquid chromatography method to measure the amount of vancomycin incorporated into DRVs was developed and validated. The details will be reported in a separate publication.

### 2.5. Characterization of Physical Stability of Liposomes

The particle size, size distribution, change in mean particle size with time, and physical appearance of the liposomal suspension are sensible indicators of the kinetic stability of liposomal suspensions. The particle size and the size distribution of both the conventional and PEGylated liposomes were measured, and 5 mL each of both the formulations was stored at 4°C, room temperature (24°C), and physiologic temperature (37°C). At specific time intervals of 1, 2, and 3 months, the samples were taken, and their physical appearance was examined. In addition, the mean particle size, size distribution, and encapsulation efficiency of both formulations were measured as described above.

## 3. Results

### 3.1. Formulation and Characterization of Liposomes

#### 3.1.1. Particle Size

Both the conventional and PEGylated liposomal formulations prepared by thin-film hydration have shown a mean particle size of 228 ± 150 nm and 103 ± 45 nm, respectively, and those prepared by ammonium sulfate gradient method have shown a mean particle size of 242 ± 87 nm and 229 ± 66 nm, respectively.

The mean particle size of conventional liposomes prepared by dehydration-rehydration method was 254 ± 147 nm and that of PEGylated liposomes was 245 ± 139 nm (Table 1). PEGylated liposomes even without extrusion through different-sized filters had lower particle size compared to conventional liposomes. The size distribution curve was uniform for both the formulations. Low standard deviation for PEGylated formulations showed narrow distribution compared to conventional liposomes. 

#### 3.1.2. Encapsulation Efficiency

Encapsulation efficiency, determined as the percentage of vancomycin incorporated into DRVs relative to the initial amount of drug added, was initially calculated spectrophotometrically at 280 nm, when the liposomes were prepared by thin-film hydration and ammonium sulfate gradient methods. The %EE of conventional liposomes prepared by thin-film hydration was 2 ± 1% and that of PEGylated formulation was 4 ± 2%, whereas the conventional and PEGylated liposomes prepared by ammonium sulfate gradient method showed a %EE of 0.3 and 0.1%, respectively. When the liposomes were prepared by dehydration-rehydration method the %EE was calculated using HPLC after lysing the liposomes in 10% triton X-100. The encapsulation efficiency of conventional liposomes was 9 ± 2% and that of PEGylated liposomes was 13 ± 3% (Table 1). There was considerable improvement in encapsulation efficiency when DRV method was used for liposomal preparation compared to those prepared by thin-film hydration and ammonium sulfate gradient methods.

### 3.2. Physical Stability of Liposomes

The physical appearance of both the conventional and PEGylated liposomal formulations stored at 4°C, 24°C, and 37°C for 1, 2, and 3 months was evaluated. At the end of 1 month, all the liposomal formulations stored at 4°C and 24°C were stable. But for the formulations stored at 37°C, a change in the color and viscosity of the liposomal suspension was observed. Growth of fungus on top of the suspension stored at 37°C was observed with both conventional and PEGylated liposomal formulations, which led to discontinuation of the stability studies at 37°C after 1 month. At the end of 2nd and 3rd months, both the formulations stored at 24°C showed fungal growth, while those at 4°C were stable.

The particle size distribution and the mean particle size of both the formulations as a function of temperature were also evaluated after the end of 1, 2, and 3 months. Except for the liposomes stored at 37°C, there is no significant change in the particle size and size distribution of other liposomal formulations stored at 4°C and 24°C (Table 2).

**Figure 2:**
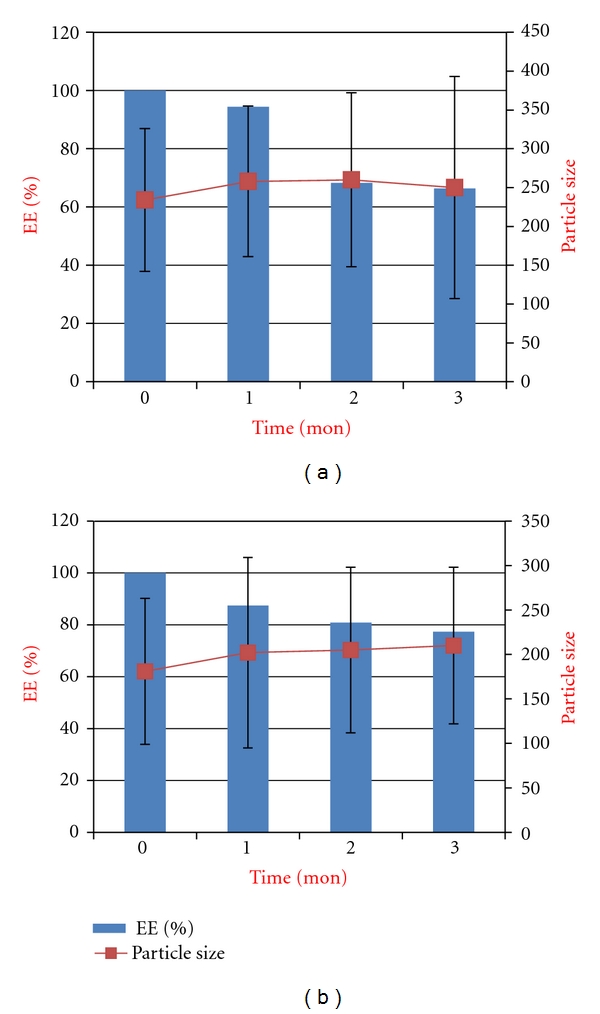
Physical stability of conventional (a) and PEGylated (b) liposomal formulations of vancomycin at 4°C by the end of 1, 2, and 3 months.

Encapsulation efficiency of the conventional liposomes stored at 4°C decreased by 6, 32, and 34%, whereas those stored at 24°C decreased by 30, 60, and 70% at the end of 1, 2 and 3 months respectively. For the PEGylated liposomes stored at 4°C, encapsulation efficiency decreased by 13, 19, and 23%, whereas for those stored at 24°C decreased by 27, 59, and 61% at the end of 1, 2, and 3 months, respectively. The results of particle size and % EE of both the formulation over the span of 3 months were shown in Figure 2.

## 4. Discussion

MRSA has become an important etiology of pneumonia both in healthcare and community settings [1]. Although considered to be an extracellular pathogen, *Staphylococcus aureus *is able to invade a variety of mammalian, nonprofessional phagocytes and can also survive engulfment by professional phagocytes such as monocytes and neutrophils [25, 26]. Thus antibiotics which penetrate these cells have been shown to have superior clinical efficacy. Vancomycin has been the cornerstone of therapy for serious MRSA-associated infections. However, vancomycin has been associated with clinical failure rates [27, 28], due to poor penetration into eukaryotic cells, which is mainly essential for eradication of intracellular bacteria. Furthermore, vancomycin tolerance is emerging as evidenced by reports of incremental increases in MIC (“MIC creep”) in MRSA isolates [29, 30]. Invasive infections due to MRSA strains with MICs ranging from 1 to 2 *μ*g/mL have lower success rates as compared to infections due to strains with MIC ≤ 0.5 *μ*g/mL [31, 32]. Pharmacodynamic studies suggest that curative vancomycin treatment of MRSA infection requires achieving a ratio of the area under the concentration time curve for 24 h to minimum inhibitory concentration (AUC_24_/MIC) ≥ 400 [33]. Higher serum vancomycin levels would therefore be needed and in turn would increase the risk of nephrotoxicity [34]. Liposomal encapsulation of certain drugs has been shown to deliver the entrapped drug into phagocytic cells leading to intracellular drug accumulation [13, 35]. We sought to construct liposomes with high yield entrapment of vancomycin as well as favorable stability, which allow potentially nephrotoxic vancomycin to be delivered in high enough concentrations in the lung to effectively kill viable tissue-invasive MRSA pathogens while sparing the host of worsening injury to the kidneys.

Both the conventional and PEGylated liposomal formulations were initially prepared by thin-film hydration method [10] and ammonium sulfate gradient method [23]. High entrapment of vancomycin using passive loading methods was not enough. The active entrapment method using a transmembrane pH gradient failed to entrap vancomycin properly because of its large molecular size and charge property. The encapsulation efficiency was very low for both the formulations, especially for PEGylated liposomes, the encapsulation was around 5%. A change in amount of vancomycin to be encapsulated from 50 mg to 100 mg did not cause any appreciable change in the final encapsulation efficiency. To further improve the encapsulation efficiency of vancomycin, a modified dehydration-rehydration method [24] was used. In this method, a suspension of empty liposomes is lyophilized. Since such lipid has a highly organized structure, addition of water with the desired solute can rehydrate, fuse, and reseal vesicles with high capture efficiency, unlike the conventional methods. As vancomycin is highly water soluble, its encapsulation is commonly correlated with the concentration of encapsulated aqueous volume. DRVs are liposomes that are formulated under mild conditions and have the capability to entrap substantially high amounts of hydrophilic solutes, compared to other types of liposomes [15]. Their high entrapment is due to the fact that preformed empty liposomes are disrupted during a freeze-drying cycle and subsequently rehydrated in the presence of a concentrated solution of the solute to be encapsulated. DSPC was used as the phospholipid for preparation of both the liposomal formulations. As DSPC has high transition temperature of 56°C, it is quite stable and is in solid state holding the drug and decreasing its release rate. Incorporation of cholesterol protects the DRVs from osmotic shock [15]. Large unilamellar liposomes (LUVs) are particularly useful for passive targeting to macrophages. LUVs are taken up by phagocytic cells more rapidly than SUVs. LUVs provide a system that allows relatively high trapping efficiency as well as relatively slow clearance. LUVs of reproducible size and homogeneity were prepared by sequential extrusion of multilamellar liposomes through polycarbonate membranes with no degradation of phospholipids.

It has been well documented for various liposomal drug formulations that the liposomes without sugar were significantly larger than those prepared with sugar [22, 36]. The ability of sugars to prevent vesicle fusion has been attributed to the formation of a stable glassy state as well as direct interaction between the polar head groups of phospholipids and sugars [37, 38]. Different types of cryoprotectants like sucrose, lactose, mannitol, and trehalose were investigated. The particle size of the liposomes appeared to be stable in the final liposomal formulations when sucrose was used as the cryoprotectant. Presence of sucrose preserved the stability of the vesicles during freeze-drying and also allowed for vesicle destabilization during rehydration, so that more vancomycin could be entrapped into the aqueous phase. However, higher initial concentrations of sucrose caused a decrease in the vancomycin entrapment. PEGylation of liposomes significantly reduces RES uptake and prolongs the blood circulation time [39], which mainly depends on both the amount of grafted PEG and the molecular weight of the polymer. A decrease in the amount of MPEG-DSPE from 6 to 4 mg caused an increase in the encapsulation efficiency. For the separation of nonencapsulated drug from encapsulated drug, centrifree filtration and gel filtration (using sepharose-4B) techniques were used initially [40]. The upper limit for lipid concentration was 5 mg/mL using the centrifree method, and separation using gel chromatography led to the dilution of the liposomal preparation. These drawbacks led to the use of ultracentrifugation technique for the separation of unencapsulated drug. The obtained liposomal preparation was lysed using triton X-100 and encapsulation efficiency was accurately determined by the HPLC method. All the prepared formulations were stored as suspensions, as lyophilizing the liposomes for the second time during the preparation did not yield a satisfactory cake. The liposomal suspensions were stable and could be able to retain the encapsulated vancomycin when stored at 4°C, which is evident from the stability experimental results.

Both the liposomal formulations prepared by the DRV method had a very narrow particle size distribution. The size of liposomes is an important determinant of encapsulation efficiency. As the size of liposomes increases, the entrapped volume increases for constant lamellarity [41]. However, larger liposomes have short circulation half-life as they are rapidly eliminated from the circulation by the RES [42]. PEG lipids reduce the requirement of small size for long circulation, but their influence is restricted within a size range. A size range of 210–275 nm is the optimum size where PEG liposomes retain their prolonged circulation [43]. Therefore a balance between the liposomal size and entrapment efficiency was maintained in case of both the conventional and PEGylated vancomycin liposomal formulations.

The stability of liposomes is another important factor to be considered in the development of an efficient drug delivery system. Therefore, we evaluated the stability of both the liposomal formulations at different temperatures, to mimic physiological conditions. The results obtained have shown that the prepared vesicles were physically and chemically stable at 4°C for 3 months. No significant change in the physical appearance, particle size, and size distribution was observed for both the formulations during the course of stability study at 4°C. But the formulations stored at 24°C and 37°C showed a slight increase in particle size, which may be due to the aggregation or swelling of liposomes. Slight decrease in the encapsulation efficiency was observed which shows a significant leakage of vancomycin from both the formulations over time.

Both conventional and PEGylated liposomal vancomycin formulations prepared by the DRV method described in this paper and stored at 4°C have been used successfully in an *in vivo* study to investigate the pharmacokinetics and biodistribution of vancomycin in various formulations. It has been demonstrated that both conventional and PEGylated liposomal formulations of vancomycin lead to enhanced biodistribution *in vivo* compared to standard vancomycin. Furthermore, PEGylated liposomal vancomycin shows improved pharmacokinetics and prolonged half-life compared to conventional liposomal formulation.

## 5. Conclusions

Both the conventional and PEGylated liposomal formulations of vancomycin were successfully prepared using a modified dehydration-rehydration method. The DRV method uses mild conditions and has the capability to entrap substantially high amounts of hydrophilic solutes, compared to other types. Both the formulations have a narrow particle size and size distribution and % encapsulation efficiency of 9 ± 2 and 12 ± 3, respectively. Addition of sucrose preserved the stability of the formulations during freeze-drying. The prepared formulations were stable in suspension form at 4°C for 3 months.

## Figures and Tables

**Table 1:** Particle size and encapsulation efficiency of conventional and PEGylated liposomes prepared using the DRV method.

Formulation	Size (nm)	% EE	Concentration (mg/mL)
Conventional liposomes	254 ± 147	9.0 ± 2.2	1.13
PEGylated liposomes	245 ± 139	12.6 ± 3.0	1.58

**Table 2:** Stability of conventional and PEGylated vancomycin liposomes at the end of 1, 2 and 3 months.

Formulation	Test condition	Temp	% EE	Particle size (nm)	Physical appearance
Conventional liposomes	0 Mon		12.40	234 ± 92	Milky suspension
	1 Mon	4°C	11.71	258 ± 111	Milky suspension
		24°C	8.68	242 ± 97	Milky suspension; slight coagulation
		37°C	4.28	605 ± 800	Slightly dark color and thicker suspension, with growth of fungus on top, dispersed upon shaking.
	2 Mon	4°C	8.47	260 ± 85	Milky suspension
		24°C	5.01	305 ± 112	Slight dark color suspension with formation of big clumps of fungus, which are not dispersible upon shaking.
	3 Mon	4°C	8.23	250 ± 97	Milky suspension
		24°C	3.67	356 ± 43	Slight dark color suspension with formation of big clumps of fungus, which are not dispersible upon shaking

PEGylated liposomes	0 Mon		12.78	181 ± 82	Milky suspension
	1 Mon	4°C	11.17	202 ± 107	Milky suspension
		24°C	9.40	200 ± 106	Milky suspension; slight coagulation
		37°C	6.90	208 ± 109	Slightly dark color and thicker suspension, with growth of fungus on top, dispersed upon shaking.
	2 Mon	4°C	10.33	205 ± 93	Milky suspension
		24°C	5.29	355 ± 154	Slight dark color suspension with formation of big clumps of fungus, which are not dispersible upon shaking.
	3 Mon	4°C	9.89	210 ± 88	Milky suspension
		24°C	4.98	290 ± 120	Slight dark color suspension with formation of big clumps of fungus, which are not dispersible upon shaking
